# *PeCLH2* Gene Positively Regulate Salt Tolerance in Transgenic *Populus alba* × *Populus glandulosa*

**DOI:** 10.3390/genes14030538

**Published:** 2023-02-21

**Authors:** Xiaolan Ge, Jiujun Du, Lei Zhang, Guanzheng Qu, Jianjun Hu

**Affiliations:** 1 State Key Laboratory of Tree Genetics and Breeding, Research Institute of Forestry, Chinese Academy of Forestry, Xiangshan Road, Beijing 100091, China; 2 State Key Laboratory of Tree Genetics and Breeding, Northeast Forestry University, Hexing Road, Harbin 150040, China; 3 Collaborative Innovation Center of Sustainable Forestry in Southern China, Nanjing Forestry University, Nanjing 210037, China

**Keywords:** *Populus euphratica*, *PeCLH2*, salt stress, physiologic indexes, transcriptome

## Abstract

Salt is an important environmental stress factor, which seriously affects the growth, development and distribution of plants. Chlorophyllase plays an important role in stress response. Nevertheless, little is known about the physiological and molecular mechanism of chlorophyll (Chlase, *CLH*) genes in plants. We cloned *PeCLH2* from *Populus euphratica* and found that *PeCLH2* was differentially expressed in different tissues, especially in the leaves of *P. euphratica*. To further study the role of *PeCLH2* in salt tolerance, *PeCLH2* overexpression and RNA interference transgenic lines were established in *Populus alba* × *Populus glandulosa* and used for salt stress treatment and physiologic indexes studies. Overexpressing lines significantly improved tolerance to salt treatment and reduced reactive oxygen species production. RNA interference lines showed the opposite. Transcriptome analysis was performed on leaves of control and transgenic lines under normal growth conditions and salt stress to predict genes regulated during salt stress. This provides a basis for elucidating the molecular regulation mechanism of *PeCLH2* in response to salt stress and improving the tolerance of poplar under salt stress.

## 1. Introduction

Due to unreasonable irrigation, environmental pollution and other factors, soil salinization is becoming more and more serious, and salt stress has become one of the important reasons to limit plant growth and restrict the development of the agricultural industry [1,2,3]. At present, about 1/5 of the arable land in China is salinized, with a total area of up to 98 million hm^2^ [4]. China is the world’s most populous country with less than 0.1 hm^2^ of arable land per capita, and it is a difficult issue to utilize the salinized land and promote the increase in food production [5]. Therefore, the study of salt tolerance and physiological mechanisms of plants and the screening and breeding of salt-tolerant crop varieties have become some of the hot spots in the field of agricultural research.

*P. euphratica* is a precious forest resource unique to desert areas. Growing in the desert all year round, it has a strong integrated resistance to salinity, barrenness, cold and drought [6,7,8,9]. Therefore, it is important to screen the excellent resistance genetic resources in *P. euphratica* and combine them with genetic engineering to breed resistant and high-quality tree species suitable for the needs of arid and semi-arid regions in China.

Chlorophyll (Chlase, CLH) is an important pigment for photosynthesis in plants, and its synthesis and degradation exist in a dynamic form in the plant. Chlorophyll degradation is closely related to plant physiological phenomena such as plant senescence, fruit ripening and tree defoliation [10,11,12]. Chlorophyllase has been of interest as the key enzyme that catalyzes this degradation reaction. Globally, about 10^9^ kg of chlorophyll is reported to be degraded in plants annually [13,14,15]. Among them, CLH is the key rate-limiting enzyme in the chlorophyll degradation process, which can hydrolyze chlorophyll into dephytolized chlorophyll and phytol of carboxylic acid [16]. It has been shown in the literature that the expression of chlorophyllase genes and changes in CLH activity in plants may change significantly under multiple exogenous stimuli (such as heavy metals, low temperature, high temperature, salinity, drought, injury, pathogens, pest infestation, salicylic acid/SA) [17,18,19,20]. It has been reported that *ERF72* can directly bind to the promoter region of the iron deficiency response gene *CLH1* and reduce CLH gene expression [21]. Overexpression of *ERF4* leads to degradation of chlorophyll content and up-regulation of the *CLH1* gene in tobacco [22]. In addition, CLH genes have been isolated successively in *Citrus reticulata* [14], *Chlamydomonas reinhardtii* [23], *Ginkgo biloba* [24] and *Chenopodium album* [25] plants by previous authors, and preliminary studies and have been conducted to find that CLH genes of different species have different expression patterns and functions during plant growth and development [26]. Despite the fact that chlorophyll controls various important traits, so far, there have been few studies on the molecular mechanisms of chlorophyll genes for salt tolerance in forest trees.

In this study, a salt-tolerant chlorophyll gene, *PeCLH2*, was identified through transcriptome analysis of *P. euphratica* in the early stage, and heterologous expression of the *PeCLH2* gene was performed in *P. alba* × *P. glandulosa* [27,28]. Transcriptome analyses were performed on overexpression, RNA interference expression and wild-type plants. Gene Ontology (GO) and Kyoto Encyclopedia of Genes and Genomes (KEGG) analyses indicated that differential genes were mainly enriched in isoquinoline alkaloid biosynthesis, tyrosine metabolism-α-linolenic acid metabolism and phytohormone signaling pathways after salt stress. The results indicated that the *PeCLH2* gene positively regulates salt tolerance in transgenic *P. alba* × *P. glandulosa*. It provides a theoretical basis for the in-depth study of its regulatory mechanism and the functional study of related genes.

## 2. Materials and Methods

### 2.1. Plant Materials

The experimental material used in this study was *P. alba* × *P. glandulosa* and *P. euphratica* asexual line histoculture seedlings from natural *P. euphratica* seed propagation in Xinjiang, preserved in the State Key Laboratory of Tree Genetics and Breeding of the Chinese Academy of Forestry. It was grown in culture flasks containing 1/2 MS medium. The culture conditions were a 16/8 h light/dark cycle with an average temperature of 25 °C.

### 2.2. Genetic Phylogenetic Analysis

Protein sequences of *CLH2* genes of nine different species were obtained from the National Center for Biotechnology Information database (http://www.ncbi.nlm.nih.gov/ (accessed on 22 January 2022)) and multiple sequence alignment was performed using clustal W [29]. Phylogenetic tree analysis was performed by MEGA 6 [30] using the neighbor-joining method.

### 2.3. Molecular Cloning of the Gene

The CLH2(POPULUS_EUPHRATICA_20786) gene coding sequence was found on the *P. euphratica* genome website to design primers, the CLH2 gene sequence was cloned from *P. euphratica*, and the coding region of *PeCLH2* was cloned into the plant expression vector PMDC32 using the Gateway vector construction method to obtain a 35S::*PeCLH2* overexpression vector; *PeCLH2* RNA interference vector (RNAi-*PeCLH2*) was obtained on pH7GWIWG2-I vector [31]; and the *PeCLH2* promoter sequence was cloned into vector PBI121 to obtain *PPeCLH2*::GUS vector and transform *P. alba* × *P. glandulosa* [32].

### 2.4. GUS Experiment

The GUS tissue staining method was carried out with reference to previous studies [33]. The 2kb promoter of *PeCLH2* was cloned into PBI121 vector to obtain *PPeCLH2::GUS* transgenic plants (Table S1).The 1-month-old transgenic plants were fixed in 90% acetone at 4 °C for 2 h and transferred to GUS staining buffer (0.2M sodium dihydrogen phosphate, 0.2 M disodium hydrogen phosphate, 2.0 mM potassium ferricyanide, 2.0 mM potassium ferricyanide, ready-to-use) for washing; then, it was transferred to GUS staining solution (GUS staining buffer with 0.2% Triton X-100) for incubation at 37 °C for 12 h, decolorized with 70% ethanol, and stored at 4 °C (the solution was changed to ethanol when it turned green). The localization of GUS signals in different tissues was observed using an Olympus microscope. GUS staining experiments contained at least 6 transgenic strains, with at least 6 replicates of each transgenic strain. GUS staining experiments were repeated at least three times.

### 2.5. Breeding of Transgenic Plants with Overexpression and RNA Interference Expression

Transgenic lines were obtained by callus transformation [34]. One-month-old transgenic resistant buds were transferred to a rooting medium containing plants cultured on 200 mg L^−1^ cephalexin and 1.5 mg L^−1^ hygromycin B. Transgenic poplars were examined at the molecular-level DNA and by real-time PCR. RT-qPCR was performed on a LightCycler 480 thermal (Roche, Basel, Switzerland) cycler using the SYBR PreMix Ex Taq kit (Dalian Takara, China) according to the manufacturer’s instructions (Table S1). The qPCR procedures were: 95.0 °C for 30 s, 95.0 °C for 5 s, 60.0 °C for 34 s, 95.0 °C for 15 s, 60.0 °C for 1 min, and 95.0 °C for 15 s. Data were processed using the 2^−ΔΔCT^ method. Each sample had at least three biological replicates.

### 2.6. Morphometry

Two high-expression lines, OC1 and OC9, were overexpressed, and RC6 and RC10 with high silencing efficiency were selected for salt stress test in RNA interference transgenic lines. For the convenience of analysis and reading, we have simplified this. OC1 is marked as OC1, OC9 as OC2, RC6 as RC1, and RC10 as RC2.Transgenic lines and wild-type (WT) seedlings in consistent growth status were transferred to 1/2 MS medium containing 0, 50, 75 and 100 mM NaCl and grown.

The plant height, root length and fresh weight of 1-month-old plants were determined. There were at least 6 individual replicates for each line, and the experiment was repeated at least 3 times.

### 2.7. Transgenic Salt Stress Experiment, Physiological Index Determination and Histochemical Staining

Two transgenic lines each with the same growth status of *PeCLH2* gene overexpression (OC1; OC2), repressed expression (RC1; RC2) and WT were transplanted into soil pots in the greenhouse for one month. They were divided into six groups on average, with three plants per strain in each group. The three groups were treated with 150 mM NaCl for seven days, and the other three groups were treated with water as control. Each treatment contained nine plants. Phenotypic changes were observed and recorded. The second and third leaves below the top were taken for DAB and NBT staining, and the fourth and sixth leaves were taken for POD, SOD, MDA and other physiological indicators. Physiological indicators and histochemical staining including peroxidase (POD), superoxide dismutase (SOD), malondialdehyde (MDA), relative conductivity, chlorophyll content, soluble sugar content, diaminobezidin3,3 (DAB), and tetranitroblue tetrazolium chloride (NBT) were determined in transgenic lines and WT according to the manufacturer’s instructions (Solarbio, Beijing, China).

### 2.8. Expression Analysis of Stress Resistance Related Genes

Transgenic and WT tissue culture seedlings grown in culture flasks for 30 days with consistent growth were extracted from leaf RNA for Quantitative Real-time PCR (RT-qPCR) analysis, and information on the primers for the internal reference primers, POD and SOD-related genes are shown in Table S1.

### 2.9. RNA Sequencing

Transgenic and WT plants grown in soil pots for one month were treated with 150 mM NaCl for 24 h. Three overexpression transgenic lines (OC), three RNA interference transgenic lines (RC), and one wild-type control top leaf (WT) were collected, immediately frozen in liquid nitrogen, and saved in a −80 °C freezer for similar evaluation and sequencing (each strain had three biological replicates). Total RNA was extracted using the plant total RNA extraction kit (TIANGEN, Beijing, China). The purity and integrity of RNA were checked by nanodrops, agarose gel electrophoresis and the Agilent Biological Analyzer 2100 system (Agilent Technologies Co. Ltd., Beijing, China).Total RNA was extracted and sent to Gene Denovo (Guangzhou, China) for sequencing using the Illumina HiSeq2000 platform. The experimental procedure for transcriptome sequencing included library construction, library quality control and up-sequencing.

### 2.10. GO and KEGG Enrichment Analyses of DEGs

This study used the *Populus trichocarpa* genome as the reference genome. The R package DESeq2 was used to identify differentially expressed genes between transgenic lines and WT with a threshold of logFC ≥ 1.5 and *q*-value ≤ 0.05 [35]. Gene Ontology (GO) enrichment analysis and visualization of results were performed using the GOseq R package [36]. Kyoto Encyclopedia of Genes and Genomes (KEGG) enrichment analysis was performed using KEGG Orthology-Based Annotation Systerm2.0 (KOBAS) [37].

Differentially expressed genes were compared to the Plant Transcription Factor Database v3.0 (Plant TFDB v3.0) database [38] to screen for differentially expressed transcription factor genes.

### 2.11. RT-qPCR Examination of RNA Sequencing Results

To verify the reliability of transcriptome sequencing data, the expression levels of 16 stress-responsive genes were analyzed by RT-qPCR (Table S1). The relative expression levels of the genes in the transgenic strains were calculated based on their expression levels in the WT strains. Statistical analysis was performed using t-test in GraphPad Prism 8 software (GraphPad Company, San Diego, CA, USA) [39]. A *p*-value < 0.05 was considered statistically significant.

## 3. Result

### 3.1. Phylogenetic Analysis and Gene Cloning

**Figure 1:**
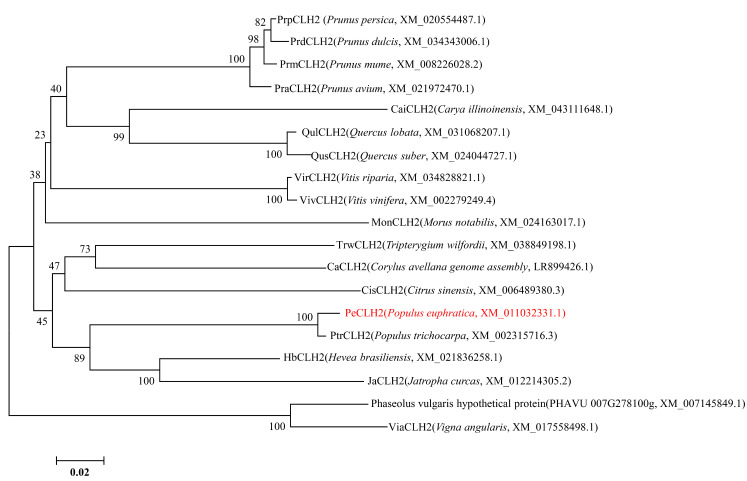
PeCLH2 protein sequence analysis of 10 plants. Full-length amino acid sequences were used for phylogenetic analysis. The phylogenetic tree was constructed using MEGA5 with the Maximum Likelihood method, 1000 repetitions of bootstrap tests, and JTT matrix-based model. The red line represents the PeCLH2.The sequences of the CLH2 proteins were obtained from the NCBI website (https://www.ncbinm.nih.gov/protein/ (accessed on 22 January 2022)), and their GenBank accession numbers are shown below. *Prunus persica* PrpCLH2 (XM_020554487.1); *Prunus dulcis PrdCLH2* (XM_034343006.1); *Prunus mume* PrpCLH2 (XM_008226028.2); *Prunus avium* PraCLH2 (XM_021972470.1); *Carya illinoinensis* CaiCLH (XM_043111648.1); *Quercus lobata* QulCLH2 (XM_031068207.1); *Quercus suber* QusCLH2 (XM_024044727.1); *Vitis riparia* VirCLH (XM_034828821.1); *Vitis vinifera* VivCLH (XM_002279249.4); *Morus notabilis* MonCLH2 (XM_024163017.1); *Tripterygium wilfordii* TrwCLH2 (XM_038849198.1); *Corylus avellana genome assembly* CaCLH2 (LR899426.1); *Citrus sinensis* CisCLH2 (XM_006489380.3); *P. euphratica* PeCLH2 (XM_011032331.1); *Populus trichocarpa* PtrCLH2 (XM_002315716.3); *Hevea brasiliensis* HbCLH2 (XM_021836258.1); *Jatropha curcas* JaCLH2 (XM_012214305.2); Phaseolus vulgaris hypothetical protein (PHAVU 007G278100g, XM_007145849.1); *Vigna angularis* ViaCLH2 (XM_017558498.1).

The cDNA fragment of *PeCLH2* was cloned from *P. euphratica*. The coding sequence region had 1005 bp and encoded 335 amino acids, the molecular weight was 83.53631 KDa, the isoelectric point was 5.08, the molecular formula was C_3068_H_5133_N_1005_O_1286_S_214_, the total number of atoms was 10,706, the fat coefficient was 28.26, the half-life was >20 h, the instability coefficient was 49.39, and the overall average hydrophilicity (GRAVY) was 0.756, and the protein was predicted to be a hydrophobic protein. The multiple sequence alignment and evolution analysis of PeCLH2 amino acid sequence were carried out by BioEdit and MEGA5.0 software. The results showed that the PeCLH2 protein sequences of 10 plants all had a conserved chlorophyll domain. The amino acid sequence identity is 78–100%. Among them, the PeCLH2 protein has high homology with the CLH2 protein of *P. trichocarpa*,(XM_002315716.3) *Hevea brasiliensis* (XM_021836258.1), and *Jatropha curcas* (XM_012214305.2) (Figure 1).

### 3.2. Expression Analysis of PeCLH2

**Figure 2:**
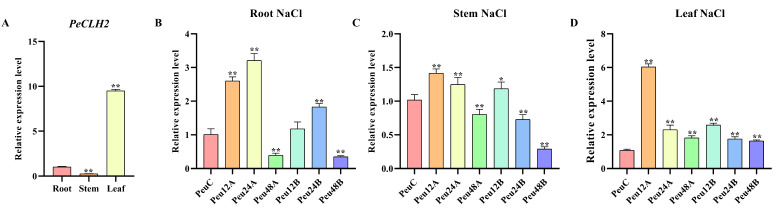
Analysis of spatiotemporal expression pattern of *PeCLH2*. (**A**). Tissue-differential expression patterns of *PeCLH2*. The expression level was calculated relative to its expression level in shoot. Three biological replicates were used. The error bars represent standard deviation. The presence of an asterisk implies that there is a substantial difference between stem, leaf and root. (**B**–**D**). Expression analysis of *PeCLH2* in response to salt stress in roots, stems, and leaves. The expression level of each gene was calculated relative to its expression level at control (PeuC). The error bars represent standard deviation. The asterisk indicates significant differences between the treatment group and the control group. Abbreviations: PeuC, *P. euphratica* control; A, 150 mM NaCl salt-stressed; B, 300 mM NaCl salt-stressed; 12, salt stress for 12 h; 24, salt stress for 24 h; 48, salt stress for 48 h (*t* test, * *p* < 0.05, ** *p* < 0.01).

To explore the expression pattern of *PeCLH2* in different tissues, we used RT-qPCR to detect its expression levels in roots, stems, and leaves in *P. euphratica*, respectively. Results indicated that *PeCLH2* had the highest expression level in leaves, followed by stems and roots. The significant differences are shown in Figure 2A.

In order to investigate the expression pattern of *PeCLH2* in *P. euphratica* under salt stress, we used RT-qPCR to determine its expression levels in roots, stems and leaves treated with 150 mM NaCl or 300 mM NaCl concentrations for 0, 12, 24, and 48 h, respectively. As shown in Figure 2B–D, the salt response of *PeCLH2* to leaves and stems segments showed an up-regulation trend from 0 h to 12 h, and then showed a gradual down-regulation trend. In addition, it is worth noting that the salt response of *PeCLH2* to *P. euphratica* root was up-regulated from 0 h to 24 h and then down-regulated.

### 3.3. Gus Staining

In order to study the function of *PeCLH2* gene promoter, the transgenic *PPeCLH2::GUS* promoter were taken out from the culture flask, the root medium was washed with running water, and GUS staining was performed. The top young parts of the transgenic plants were stained blue, indicating that the *PPeCLH2::GUS* promoter was active and could drive the expression of the GUS gene in the young tissues of the plants (Figure S1).

### 3.4. Identification of Transgenic Plants

The expression of foreign genes was detected at the transcriptional level by RT-qPCR. The real-time monitoring of the entire PCR process by the accumulation of fluorescent signals showed that the expression of fluorescent signals was detected in each gene transgenic line (Figure S2). The results showed that 35S::*PeCLH2* (OC) and RNAi-*PeCLH2* (RC) transgenic plants were expressed at the RNA level, and the relative expression levels were different.

### 3.5. Morphological Characteristics of Transgenic Plants

**Figure 3:**
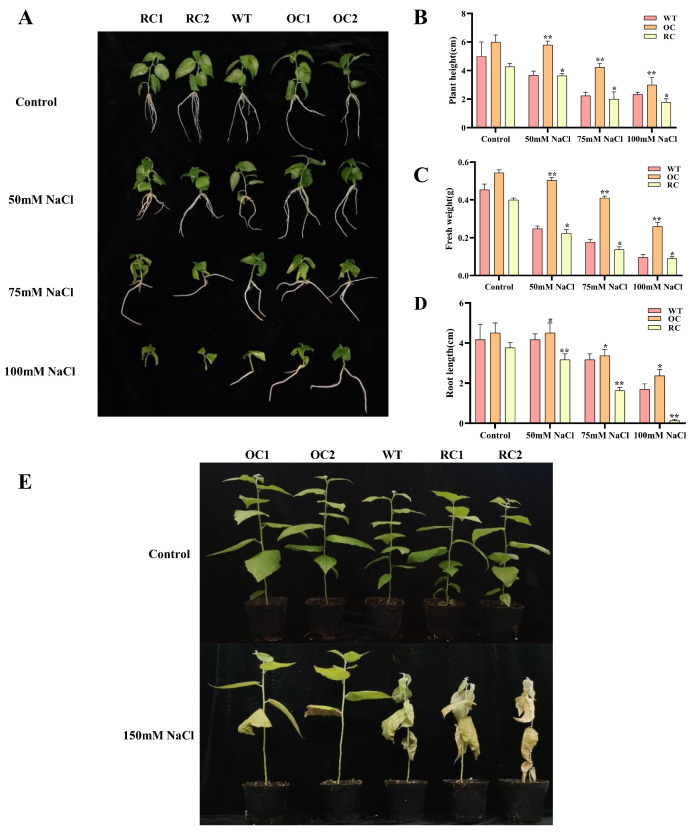
Transgenic *P. alba* × *P. glandulosa* morphological traits under salt stress. OC1, OC2, RC1, RC2: various transgenic lines; WT, wild type. (**A**–**D**) One-month-old *P. alba* × *P. glandulosa* phenotypes on 0, 50, 75, and 100 mM NaCl rooting media. Under salt stress, the height, root length, and fresh weight were measured in transgenic and WT. (**E**) The WT and transgenic plants at one-month-old were treated with respective 150 mM salt for 7 days. The standard deviation is shown by the error bar. The presence of an asterisk implies that there is a substantial difference between transgenic and WT (*t* test, * *p* < 0.05, ** *p* < 0.01).

To investigate the salt tolerance of *PeCLH2*, seedlings of 35S::*PeCLH2*, RNAi-*PeCLH2* and WT were treated with 0, 50, 75 and 100 mM NaCl, respectively. The results showed that the plant height, root length and fresh weight in the 35S::*PeCLH2* transgenic lines are superior than that of the WT at control and stress conditions. RNAi-*PeCLH2* shows the opposite situation (Figure 3A–D).

Under normal conditions, the plant height, root length and fresh weight of 35S::*PeCLH2* values were 1.2, 1.08 and 1.2 times than those of WT, respectively. RNAi-*PeCLH2* values was 0.85, 0.94 and 0.88 times, respectively. Under the condition of 50 mM NaCl, 35S::*PeCLH2* values were 1.58, 1.08 and 2 times higher than WT, respectively. RNAi-*PeCLH2* was 0.9, 0.76 and 0.7 times, respectively. Under the condition of 75 mM NaCl, 35S::*PeCLH2* values were 1.81, 1.06 and 2.3 times, respectively. RNAi-*PeCLH2* was 0.85, 0.51 and 0.77 times higher than WT, respectively. Under the condition of 100 mM NaCl, 35S::*PeCLH2* values were 1.28, 1.39 and 2.7 times, respectively. RNAi-*PeCLH2* was 0.75, 0.08, and 0.9 times higher than WT, respectively.

To investigate the role of *PeCLH2* in salt stress, we treated transgenic and wild-type plants for 7 days, which were grown in soil for one month at a NaCl concentration of 150 mM. As shown in Figure 3E, under control conditions, 35S::*PeCLH2* transgenic lines showed better growth compared to WT, while RNAi-*PeCLH2* transgenic lines did not grow differently from WT. Under salt stress, 35S::*PeCLH2* transgenic plants grew normally and showed greater salt tolerance. The WT and RNAi-*PeCLH2* transgenic appeared to wilt and show intolerance, and RNAi-*PeCLH2* transgenic line leaves wilted completely and even died. This evidence suggests that *PeCLH2* plays a crucial role in salt tolerance.

### 3.6. Physiological Index Determination and Histochemical Staining Analysis

**Figure 4:**
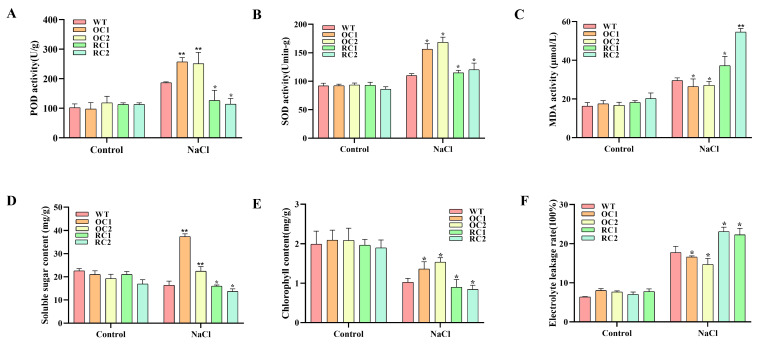
Analysis of physiology and gene expression in response to salt stress. OC1, OC2, RC1, RC2: different transgenic lines; WT, wild type. (**A**–**F**) POD, SOD, MDA, soluble sugar, chlorophyll, and relative conductivity levels were compared between transgenic and WT; The control is water. The presence of an asterisk implies that there is a substantial difference between transgenic and WT (*t* test, * *p* < 0.05, ** *p* < 0.01).

Under salt-stress conditions, the POD and SOD activities, soluble sugar content and chlorophyll content of 35S::*PeCLH2* were significantly higher than the WT, while RNAi-*PeCLH2* was significantly lower than WT. the results of MDA content and relative conductivity showed that 35S::*PeCLH2* were significantly less damaged than the WT under salt stress, while RNAi-*PeCLH2* was significantly higher than the WT (Figure 4A–E).

**Figure 5:**
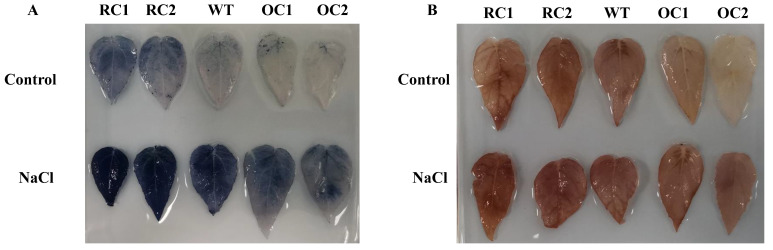
Staining with histochemical agents. (**A**) DAB staining of hydrogen peroxide. (**B**) NBT superoxide staining; WT, wild type; OC, transgenic lines with 35S: *PeCLH2* overexpression. RC, RNAi-*PeCLH2* transgenic lines.

Transgenic plants with *PeCLH2* under salt-stress conditions were stained for NBT and DAB histochemistry (Figure 5A,B). The results showed that the staining levels of leaves of transgenic plants were similar to the WT under normal conditions, indicating that there was no significant difference in the accumulation of O^2−^ and H_2_O_2_. The staining level of 35S::*PeCLH2* under salt-stress conditions was significantly lower than the WT, while RNAi-*PeCLH2* was significantly higher than the WT.

The above results showed that the damage of 35S::*PeCLH2* transgenic plants under salt stress was significantly higher than that of WT, and the damage of RNAi-*PeCLH2* under salt stress was significantly lower than WT, indicating that *PeCLH2* has certain salt tolerance.

### 3.7. Expression Analysis of Stress Resistance Related Genes

**Figure 6:**
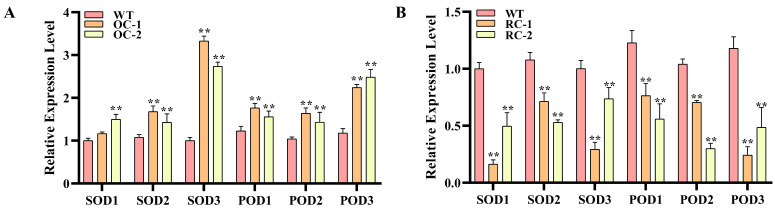
RT-PCR detection of stress-resistance-related genes. (**A**): Relative expression level of stress-resistance-related genes in *PeCLH2* transgenic lines. (**B**): Relative expression level of stress-resistance-related genes in RNAi-*PeCLH2* transgenic lines. SOD1-3: Superoxide dismutase related genes. POD1-3: Peroxidase related genes. The presence of an asterisk implies that there is a substantial difference between transgenic and WT (*t* test, ** *p* < 0.01).

The quantitative results showed that the relative expression level of stress-resistance-related genes in transgenic lines was significantly higher than WT. The relative expression levels of SOD- and POD-related genes in 35S::*PeCLH2* were 1.16–3.42 times and 1.43–2.49 times higher than WT, respectively. The relative expression levels of SOD- and POD-related genes in RNAi-*PeCLH2* were 0.16–0.74 times and 0.24–0.76 times higher than those in the WT, respectively (Figure 6A,B).

### 3.8. Sequencing Data Quality Assessment

Transcriptome analysis of WT and transgenic lines was conducted using RNA-seq technology. After the sequencing data quality control was completed, clean data were evaluated. The Q20 value of all sample data reached more than 97.37%, the Q30 value (base misjudgment rate of 0.1%) reached more than 92.73%, the GC content was between 43.73% and 44.63%, and the sequencing depth was more than ten times (Table S2). It shows that transcriptome sequencing has obtained high-quality clean data, which can meet the requirements of subsequent analysis.

### 3.9. Gene Expression Level Analysis

**Figure 7:**
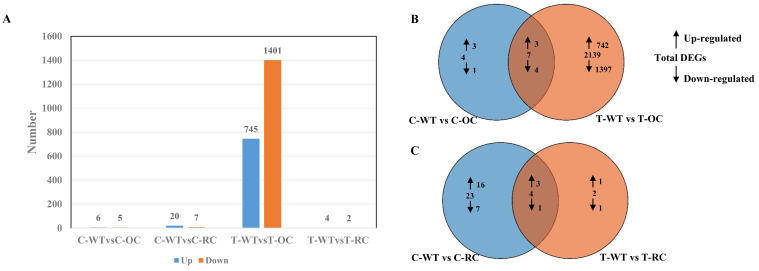
Statistics on gene expression differences between transgenic and WT before and after salt stress. (**A**) In all comparison combinations, the number of up-regulated and down-regulated genes. (**B**) Overlap of comparison C-WT vs. C-OC and T-WT vs. T-OC, of up-regulated or down-regulated genes after NaCl treatment. (**C**) Overlap of comparison C-WT vs. C-RC and T-WT vs. T-RC of up-regulated or down regulated genes after NaCl treatment.

Under normal condition, almost no significant differences were found between WT and transgenic line in terms of gene expression of the stress-related genes. However, when treated with salt, a lot of differentially expressed genes were observed in both WT and transgenic plants (Figure 7A–C). Based on the difference analysis results, we screened genes with FDR < 0.05 and |log2FC| > 1 as significantly different genes. Compared with WT, 11 differentially expressed genes (including 6 up-regulated genes and 5 down-regulated genes) were identified from 35S::*PeCLH2* (OC) in the control group. A total of 27 differentially expressed genes (including 20 up-regulated genes and 7 down-regulated genes) were identified in RNAi-*PeCLH2* (RC). In the treatment group, 2146 differentially expressed genes (including 745 up-regulated genes and 1401 down-regulated genes) were identified in OC. Six differentially expressed genes (including four up-regulated genes and two down-regulated genes) were identified in RC (Table S3). The results showed that OC and WT had the most differential genes after salt treatment, indicating that OC mainly functions after salt stress.

### 3.10. GO and KEGG Enrichment Analyses of DEGs

To determine the most important biochemical metabolic pathways and signaling pathways involved in differential gene expression in transgenic plants during salt stress, we performed GO and KEGG enrichment analysis. Global functional analysis of DEGs was performed on GO annotations into biological processes, molecular functions, and cellular components. GO enrichment analysis of all comparison groups revealed that the most common categories in the ontology of biological processes were “metabolic processes” and “single organism processes”, followed by “cellular process”. Within the category of molecular function ontology, “catalytic activity” and “binding” acted as two primary functional groups. Within the cellular component, “cell”, “cell part” and “organelle” were predominant (Figures S3 and S4).

**Figure 8:**
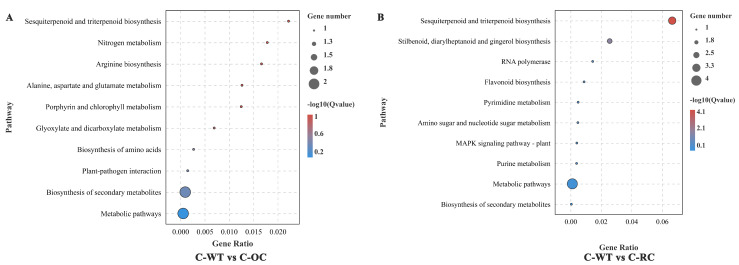
KEGG enrichment analysis of untreated transgenic transcriptome DEGs. (**A**): KEGG enrichment of the significant gene expression difference between the transgenic with expression of the *PeCLH2* overexpression and the WT under normal conditions. The enrichment analysis was performed for compartmentalization; (**B**): KEGG enrichment of the significant gene expression difference between the transgenic with RNAi-*PeCLH2* and the WT under normal conditions.

In addition, prediction of the biochemical pathways associated with the DEGs was performed by the Kyoto Encyclopedia of Genes and Genomes (KEGG) identifiers. In the untreated condition, WT and OC were enriched into 10 KEGG pathways. Sesquiterpenoid and triterpenoid biosynthesis, nitrogen metabolism, arginine biosynthesis, alanine, aspartate and glutamate metabolism, and porphyrin and chlorophyll metabolism were all significant enrichment pathways (Figure 8A). WT and RC were enriched into 10 KEGG pathways, and sesquiterpenoid and triterpenoid biosynthesis, stilbenoid, diarylheptanoid and gingerol biosynthesis RNA polymerase, flavonoid biosynthesis, and pyrimidine metabolism were significant enrichment pathways (Figure 8B). In conclusion, the biosynthesis of semi terpenoids and triterpenoids is the most abundant pathway in the untreated condition.

**Figure 9:**
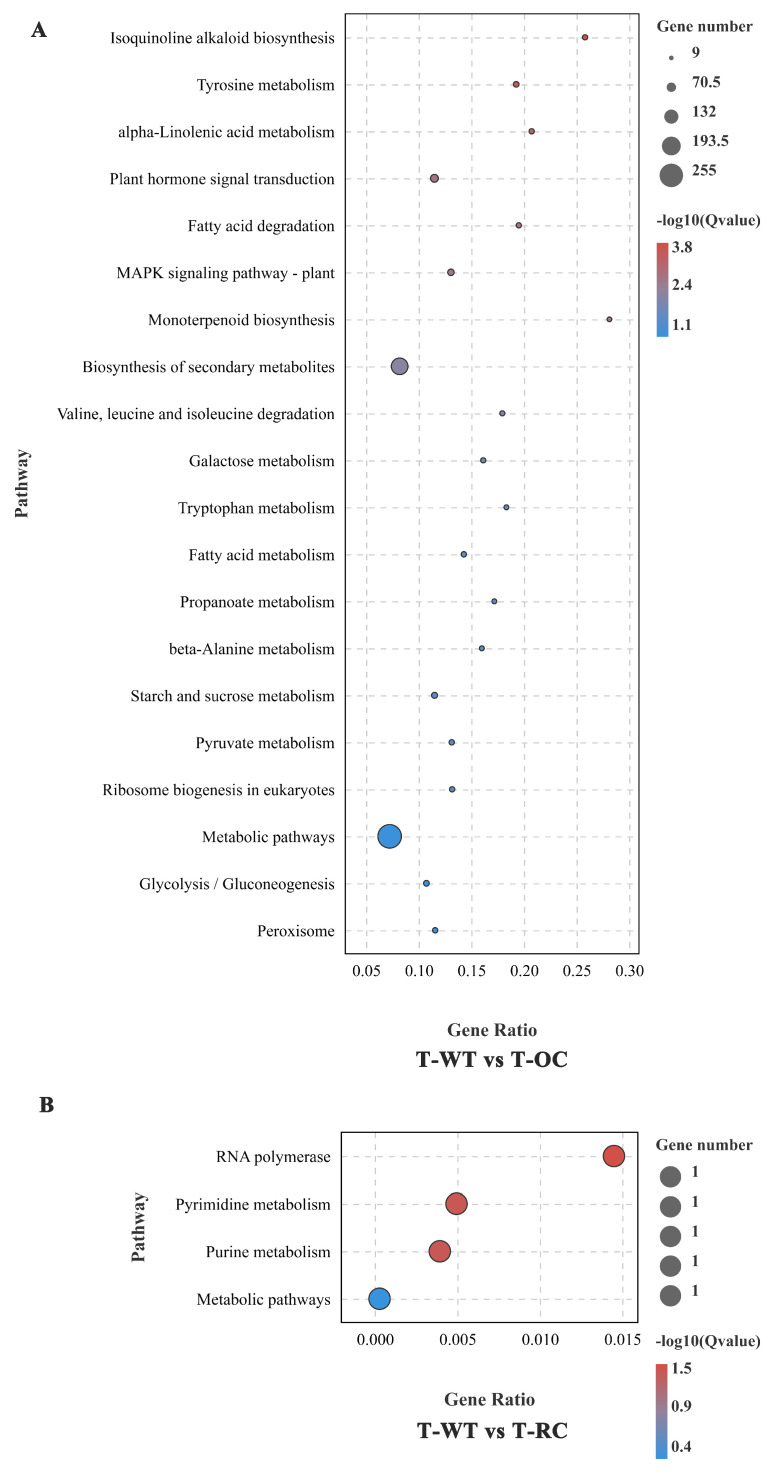
KEGG enrichment analysis of salt-treated transgenic transcriptome DEGs. (**A**): GO enrichment of the significant gene expression difference between the transgenic plants with expression of the *PeCLH2* overexpression and the WT under salt stress. The enrichment analysis was performed for compartmentalization; (**B**): KEGG enrichment of the significant gene expression difference between the transgenic with expression of the RNAi-*PeCLH2* and the WT under salt stress.

Under salt treatment, WT and OC were enriched in 116 KEGG pathways. Isoquinoline alkaloid biosynthesis, tyrosine metabolism, alpha-Linolenic acid metabolism, plant hormone signal transduction metabolic pathway, biosynthesis of secondary metabolites, and fatty acid degradation were significant enrichment pathways (Figure 9A). Among them, Isoquinoline alkaloid biosynthesis was significantly enriched with 16 differential genes, followed by tyrosine metabolism with 21 differential genes and alpha-Linolenic acid metabolism with 17 differential genes. Under salt treatment, WT and RC were enriched into four KEGG pathways, and RNA polymerase, pyrimidine metabolism, purine metabolism and metabolic pathway were significant enrichment pathways (Figure 9B). Taken together, these results suggest a fundamental change in the gene function/metabolic network in the expression of *PeCLH2* under normal and NaCl stress conditions.

### 3.11. Transcription Factors Involved in Salt Stress Response

The response of PeCLH2 transgenic plants to salt stress is synergistically regulated by multiple genes, among which transcription factors play an extremely important role. Therefore, further analysis of transcription factor differentially expressed genes in this study revealed that a total of 90 differentially expressed transcription factors, including various types of WRKY, MYB, NAC, and bHLH, were annotated under salt-stress conditions (Table S4). It is hypothesized that the effect of salt-stress treatment on transgenes was mainly concentrated on WRKY, MYB, NAC, and bHLH transcription factor families. This shows that the expression of these transcription factors is regulated by salt stress.

### 3.12. Reliability of Transcriptome Sequencing Data

**Figure 10:**
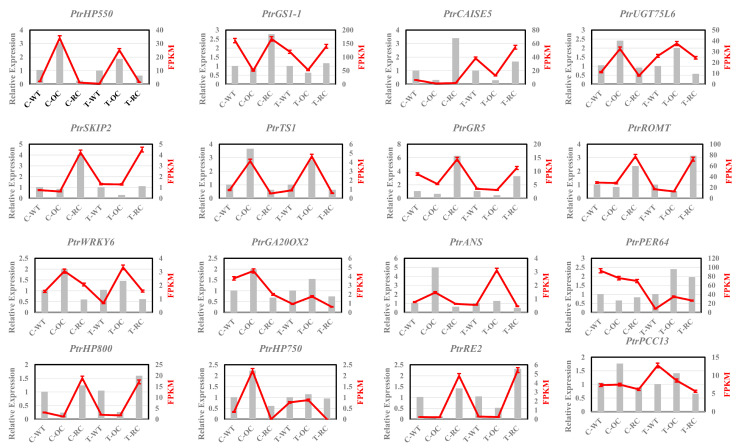
Sixteen genes were selected for validation through RT-PCR. Expression in WT plants was assigned a value of 1. The left gray histogram represents the RT-qPCR results. The red broken line on the right represents the FPKM (Fragments Per Kilobase of exon model per Million mapped fragments). Values are presented as means ± SD of three independent measurements.

To verify the accuracy of the transcriptome sequencing results, we selected 16 genes related to stress and designed primers within their conserved structural domains for RT-PCR experiments, and later compared them with the transcriptome results, which are shown in the figure below, and the results shown in the figure are almost identical to the transcriptome sequencing results (Figure 10). This indicates that the results of transcriptome data analysis are reliable.

## 4. Discussion

Chlorophyllase is one of the earliest discovered plant enzymes and one of the most important pigments in the biological world [40,41]. Chlorophyll degradation occurs when plants are subjected to biotic or abiotic stresses such as low temperature, strong light, pest and disease infestation and during plant senescence, resulting in a series of physiological responses, such as leaf fading, wilting, yellowing and premature end of life cycle [42,43,44]. *CLH* genes have been studied in plants, for example, *CLH1* and *CLH2* were identified as candidate genes by genome-wide association analysis (GWAS) of 107 *Arabidopsis* accessions, and loss of function or down-regulation of *CLH1* and *CLH2* expression promoted tolerance to the polyamine oxidase inhibitor guazatine [45,46]. Metabolomic and transcriptomic analyses of cabbage 1-methylcyclopropene treatment revealed significant inhibition of the expression of most Chl degradation-related genes (*BcPPH1/2*, *BcSGR1/2*, *BcPAO*, *BcNYC1*, and *BcNOL*) [47]. Chlorophyllase genes associated with Chl degradation were found to be up-regulated in yellow leaves in the transcriptome analysis of yellow leaf coloration in *Populus deltoides Marsh* [48]. Although CLH was discovered more than 100 years ago [49] and many studies have focused on its function and related mechanisms, little research has been reported on CLH in poplar for salt stress.

*P. euphratica* is a tall tree mainly distributed in extreme saline and semi-desert areas of Xinjiang, Gansu, Inner Mongolia in China. The strong salt tolerance of this species makes it a good model plant for studying salt tolerance, mining salt resistance genes and improving saline lands. However, the underlying molecular mechanisms controlling salt stress tolerance still have not been identified. In our previous study, we performed transcriptome analysis of salt-treated *P. euphratica* and screened a series of key salt-tolerance regulatory genes by combined WGCNA and GWAS analysis and selected the *PeERF1* and *PeCLH2* gene for functional validation. *PeERF1* has been identified for salt tolerance. *PeCLH2* is a chlorophyll family gene cloned from *P. euphratica*. Transgenes were obtained for 35S::*PeCLH2* and RNAi-*PeCLH2* gene. Morphological measurements, physiological characterization and histochemical analysis of transgenic *P. alba* × *P. glandulosa* in salt assays showed that 35S::*PeCLH2* enhanced salt tolerance in transgenic plants and that RNAi-*PeCLH2* was sensitive to salt treatment, demonstrating that *PeCLH2* plays an important role in salt-stress response.

Under high salt concentrations, antioxidant systems such as SOD and POD can scavenge ROS, and hence avoid the build-up of oxidative damage in plants to improve salt tolerance [50]. As the duration of stress increased, membrane lipid peroxidation was enhanced and MDA content increased [51]. Salt accumulation in plants disrupts cell membrane permeability, leading to an increase in conductivity; meanwhile, the content of osmoregulatory substances such as soluble sugar increases, scavenging free radicals and reducing the salt damage to plants. In this study, the changes in POD and SOD activities, MDA content, soluble sugar content, chlorophyll content and relative conductivity of transgenic poplar showed that the *PeCLH2* gene could significantly enhance salt tolerance. In addition, the up-regulation of SOD, POD, and other stress-related genes in transgenic plants 35s::*PeCLH2* and the decrease in SOD, POD, and other stress-related genes in RNAi-*PeCLH2* transgenic plants indicated that this gene enhanced salt tolerance of poplar by regulating the expression of stress-related genes.

RNA-seq data analysis showed that the overall gene expression profile of the over-transgenic plants was different from that of the control plants. Compared with the WT, 11 genes in the overexpressed transgenic lines showed significantly different expression levels before salt treatment, of which 6 were up-regulated and 5 were down-regulated. After 24 h of salt treatment, the number of DEGs in overexpressing transgenic plants increased to 2146, of which 745 were up-regulated and 1401 were down-regulated, suggesting that CLH2 may regulate many stress-related genes. Interestingly, there were 27 RNAi-*PeCLH2* transgenic lines (20 up-regulated and 7 down-regulated DEGs) before salt stress and only 6 DEGs (4 up-regulated and 2 down-regulated DEGs) after stress, and the changes in the RNAi-*PeCLH2* transgenic lines before and after salt stress were not significant. This may be due to the changes in *PeCLH2* expression levels before and after salt stress, resulting in different patterns of genetic regulation in transgenic plants.

Isoquinoline alkaloids are major secondary metabolites in a variety of plants, and the biosynthetic pathways of various isoquinoline alkaloids have been clarified through previous studies [52,53,54,55]. In the present study, the “isoquinoline alkaloid synthesis” pathway ranked first among the top 20 KEGG pathways of DEGs identified in 35S::*PeCLH2* and WT salt stress, indicating that the *PeCLH2* gene could respond to salt stress by regulating the isoquinoline biosynthesis pathway in transgenic plants (Figure 8A).

Among the downstream genes regulated by *PeCLH2*, there are many stress-resistance-related genes that play important roles in plant response to abiotic stresses. Among them, *PtrANS*, *PtrPER64*, and *PtrWRKY*6 genes are important genes that are significantly up-regulated in expression (Table S5). It has been demonstrated that *PtrANS*, *PtrPER64* and *PtrWRKY*6 genes play an important role in protecting plants from abiotic stress damage. In plants, *PtrANS*, *PtrPER64* and *PtrWRKY*6 genes can significantly improve their tolerance to abiotic stresses such as salt, osmotic and drought [56,57,58,59,60]. However, whether *PeCLH2* can directly bind to the promoters of *PtrANS*, *PtrPER64,* and *PtrWRKY*6 needs further validation.

To date, most studies on *CLH2* resistance have focused on leaf senescence, fruit ripening and chlorophyll degradation. In this study, the salt tolerance of *PeCLH2* was verified by transgenic experiments. This will provide a basis for the direction of *PeCLH2* gene resistance. In addition to the salt tolerance of *PeCLH2* gene to poplar in this study, it may also have other resistances, and further studies to verify its resistance need to be continued in the future.

## 5. Conclusions

In this study, we cloned a key regulatory gene for salinity tolerance in *P. euphratica* and *CLH2* and then transformed it in *P. alba* × *P. glandulosa*. The overexpression transgenic lines showed morphological, physiological and biochemical advantages compared to WT, while the repressed expression showed the opposite. In addition, POD and SOD related stress tolerance genes expression levels were significantly higher in the transgenic lines than in WT, indicating that *CLH2* gene could improve salt tolerance of transgenic poplars by regulating the expression of stress related genes. Many genes related to salt tolerance were identified by salt-stress transcriptome analysis of the transgenic lines, and CLH2 functioned mainly after salt stress. A series of salt-stress-related resistance genes were screened to provide candidate genes and theoretical basis for genetic engineering breeding in forest trees.

## Data Availability

Additional data supporting the findings in this study are available in the Supplementary Information of this article. The raw datasets are available from the first author or corresponding author on reasonable request.

## Supplementary Materials

The following supporting information can be downloaded at: https://www.mdpi.com/article/10.3390/genes14030538/s1. Figure S1. Promoter transgenic *P alba* × *P glandulosa* analysis of PeCLH2. Image of GUS activity assay of *PPeCLH2::GUS* transformed *P alba* × *P glandulosa*. Scale bar is 5 mm. Figure S2. PeCLH2 expression analysis in transgenic *P. alba* × *P. glandulosa*. (A) and (B), Analysis of *PeCLH2* expression levels in various transgenic strains. The expression level of *PeCLH2* was estimated in relation to that of the wild-type plant. WT, wild type poplar; OC1-32, *PeCLH2* overexpression lines; RC1-10, RNAi-*PeCLH2* expression lines. The expression level of each line was calculated relative to its expression level at WT (*t* test, *p* < 0.05). Figure S3. Gene ontology (GO) term analysis of untreated transgenic transcriptome DEGs. A: GO enrichment of the significant gene expression difference between the transgenic with expression of the *PeCLH2* overexpression and the WT under normal conditions. The enrichment analysis was performed for compartmentalization; B: GO enrichment of the significant gene expression difference between the transgenic with expression of the RNAi-*PeCLH2* and the WT under normal conditions. Figure S4. Gene ontology (GO) term analysis of salt-treated transgenic transcriptome DEGs. A: GO enrichment of the significant gene expression difference between the transgenic with expression of the *PeCLH2* overexpression and the WT under salt stress. The enrichment analysis was performed for compartmentalization; B: GO enrichment of the significant gene expression difference between the transgenic with expression of the RNAi-*PeCLH2* and the WT under salt stress. Table S1. The primers used in this study. Table S2. Statistics of *PeCLH2* transgenic transcriptome sequencing data comparison results. Table S3. DEGs identification of *PeCLH2* transgenic for the salt stress transcriptome. Table S4. Differential expression of TFs after salt stress. Table S5. Differentially expressed genes related to stress.
