# Supplementary figures and images for: PeCLH2 Gene Positively Regulate Salt Tolerance in Transgenic Populus alba × Populus glandulosa
Source: Genes (Basel). 2023 Feb 21;14(3):538. doi: 10.3390/genes14030538 (PMC10048402; doi:10.3390/genes14030538)

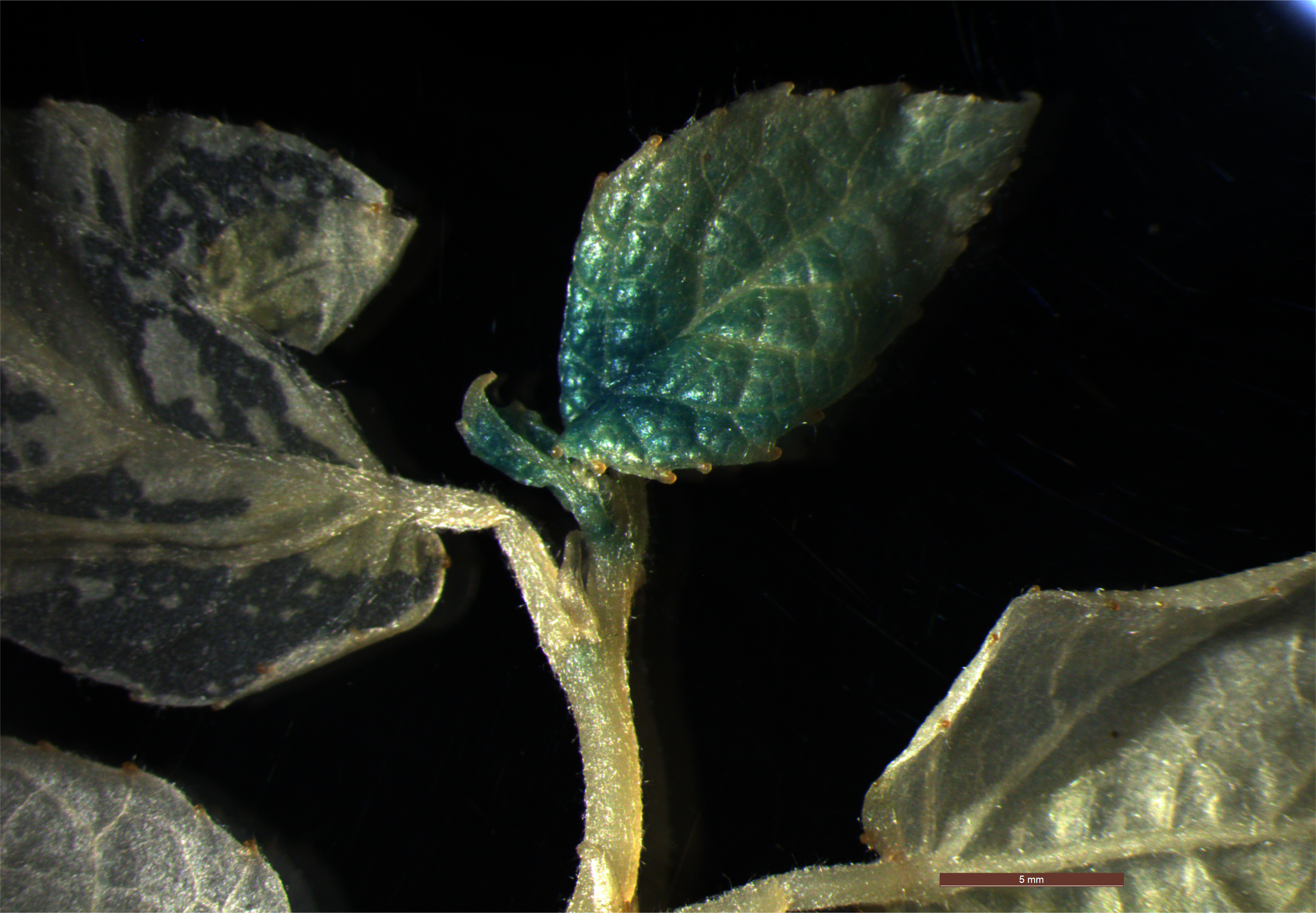

Supplement: Supplementary file 1 [file genes-14-00538-s001.zip › Figure S1.jpg]

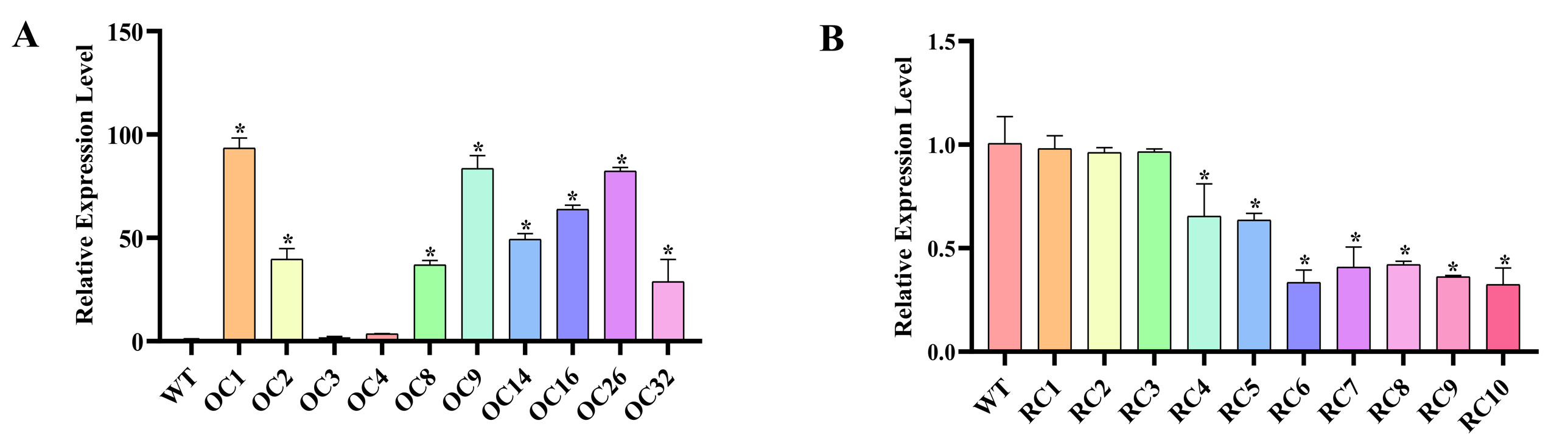

Supplement: Supplementary file 1 [file genes-14-00538-s001.zip › Figure S2.jpg]
